# Isolation, Identification, and Biocontrol Potential of Entomopathogenic Nematodes and Associated Bacteria against *Virachola livia* (Lepidoptera: Lycaenidae) and *Ectomyelois ceratoniae* (Lepidoptera: Pyralidae)

**DOI:** 10.3390/biology11020295

**Published:** 2022-02-11

**Authors:** Saqer S. Alotaibi, Hadeer Darwish, Madiha Zaynab, Sarah Alharthi, Akram Alghamdi, Amal Al-Barty, Mohd Asif, Rania H. Wahdan, Alaa Baazeem, Ahmed Noureldeen

**Affiliations:** 1 Department of Biotechnology, College of Science, Taif University, P.O. Box 11099, Taif 21944, Saudi Arabia; hadeer@tu.edu.sa; 2 Shenzhen Key Laboratory of Marine Bioresource & Eco-Environmental Sciences, College of Life Sciences and Oceanography, Shenzhen University, Shenzhen 518107, China; madiha.zaynab14@gmail.com; 3 Department of Chemistry, College of Science, Taif University, P.O. BOX 11099, Taif 21944, Saudi Arabia; sarah.alharthi@tu.edu.sa; 4 Department of Biology, College of Science, Taif University, P.O. Box 11099, Taif 21944, Saudi Arabia; a.alghamdii@tu.edu.sa (A.A.); aalbarty@tu.edu.sa (A.A.-B.); aabaazeem@tu.edu.sa (A.B.); a.noureldeen@tu.edu.sa (A.N.); 5 Regional Ayurveda Research Institute (CCRAS), Ranikhet 263645, Uttarakhand, India; asifgc2616@gmail.com; 6 Agricultural Research Center, Department of Nematode Diseases Research, Plant Pathology Research Institute, Giza 12619, Egypt; d.rwahdan44@gmail.com

**Keywords:** entomopathogenic nematodes, entomopathogenic bacteria, *Virachola livia*, *Ectomyelois ceratoniae*, biological control

## Abstract

**Simple Summary:**

For sustainable agriculture, there is a need to identify and evaluate more biocontrol agents, including entomopathogenic nematodes (EPNs). In this study, EPNs and their associated entomopathogenic bacteria symbionts (EPBs) were isolated and identified from 320 soil samples collected in Taif, Saudi Arabia. The biocontrol efficacy of EPNs and EPBs was also evaluated against third instar larvae of the pomegranate butterfly, *Virachola livia,* and the carob moth, *Ectomyelois ceratoniae*, two important insect pests of pomegranate, under laboratory conditions. Our results show that the EPNs *Steinernema* spp. were more virulent than *Heterorhabditis* spp. against the two pomegranate insects. In addition, the EPB *Stenotrophomonas maltophilia* CQ1, isolated from *Steinernema* spp., surpassed *Pseudomonas mosselii* SJ10, associated with *Heterorhabditis* spp., in their ability to kill *V. livia* or *E. ceratoniae* larvae. We conclude that either application of EPNs’ infective juveniles (IJs) or their associated EPBs could serve as potential biocontrol agents for *V. livia* and *E. ceratoniae*.

**Abstract:**

*Virachola livia* (Lepidoptera: Lycaenidae) and *Ectomyelois ceratoniae* (Lepidoptera: Pyralidae) are the key pests of pomegranates in Saudi Arabia that are managed mainly using broad-spectrum pesticides. Interactions between the entomopathogenic nematodes (EPNs) Steinernematids, and Heterorhabditids, and their entomopathogenic bacterial symbionts (EPBs) have long been considered monoxenic 2-partner associations responsible for killing insects and, therefore, are widely used in insect pest biocontrol. However, there are limited reports identifying such organisms in Taif, Saudi Arabia. The current study aimed to identify the EPNs and their associated bacteria isolated from Taif, Saudi Arabia, and evaluate their biocontrol potential on third instar larvae of *V. livia* and *E. ceratoniae* under laboratory conditions. A total of 35 EPN isolates belonging to *Steinernema* (20) and *Heterorhabditis* (15) were recovered from 320 soil samples. Twenty-six isolates of symbiotic or associated bacteria were isolated from EPNs and molecularly identified as *Xenorhabdus* (6 isolates), *Photorhabdus* (4 isolates), *Pseudomonas* (7), or *Stenotrophomonas* (9). A pathogenicity assay revealed that *Steinernema* spp. were more virulent than *Heterorhabditis* spp. against the two pomegranate insects, with LC_50_ values of 18.5 and 13.6 infective juveniles (IJs)/larva of *V. livia* for *Steinernema* spp. and 52 and 32.4 IJs/larva of *V. livia* for *Heterorhabditis* spp. at 48 and 72 h post-treatment, respectively. Moreover, LC_50_ values of 9 and 6.6 IJs/larva (*Steinernema* spp.) and 34.4 and 26.6 IJs/larva (*Heterorhabditis* spp.) were recorded for *E. ceratoniae* larvae at 48 and 72 h post-treatment. In addition, the EPB *Stenotrophomonas maltophilia* CQ1, isolated from *Steinernema* spp., surpassed *Pseudomonas mosselii* SJ10, associated with *Heterorhabditis* spp., in their ability to kill *V. livia* or *E. ceratoniae* larvae within 6 h post-application, resulting in 100% mortality in both insects after 24 and 48 h of exposure. We conclude that either application of EPNs’ IJs or their associated EPBs could serve as potential biocontrol agents for *V. livia* and *E. ceratoniae*.

## 1. Introduction

Nematodes that parasitise insects, known as entomopathogenic nematodes (EPNs), have been identified within 23 nematode families [1]. For several years, EPNs belonging to the Steinernematidae and Heterorhabditidae (Rhabditida) families have received the most attention as potential biocontrol agents because of their wide host spectrum, active host-seeking, easy mass production, low cost, long-term efficacy, easy application, compatibility with most chemicals, environmental safety for humans and other non-target organisms, capacity to reduce pesticide residues in food, and ability to increase activity of other natural enemies and increase biodiversity in managed ecosystems [2]. *Photorhabdus* and *Xenorhabdus* are Gram-negative bacteria of the family Enterobacteriaceae and are symbiotically associated with the entomopathogenic nematodes *Heterorhabditis* and *Steinernema*, respectively [3]. The infective juveniles (IJs) of Steinernematid and Heterorhabditid nematodes carrying symbiotic bacteria in their midguts live in the soil of diverse ecological systems [4]. These nematodes actively seek insect hosts, penetrating through an insect’s mouth, anus, or spiracles to reach the hemocoel, where symbiotic bacteria (e.g., *Xenorhabdus* spp. and *Photorhabdus* spp.) are released [5]. Subsequently, the symbiotic bacteria colonise the insect haemolymph, degrade insect tissues, and produce several immunosuppression factors, including toxin complexes, hydrolytic enzymes, and haemolytic and antimicrobial compounds that kill insect hosts, typically within 48 h [6,7]. Finally, the symbiotic bacteria replicate rapidly and cause septicaemia in insects [8]. This process converts the insect cadaver into suitable food for nematode development and reproduction. However, several studies have cast doubt on this view because some entomopathogenic pairs have been found to have attenuated virulence or to be non-virulent when injected alone into insects [9,10,11]. EPNs have been used against soil pests such as white grubs, root weevils, rootworms, sciarid flies, cutworms, and armyworms [12]. Satisfactory results concerning the effects of EPNs and/or EPBs in the laboratory and under field conditions have been recorded for controlling cherry fruit flies and mosquitoes as well as the pomegranate aphid, cabbage worm, and scarab beetle [13,14,15]. The protease inhibitor protein encoded gene from strains BJFS526 and Xbpi-1 of the symbiotic bacterium *Xenorhabdus bovienii* has been identified and expressed. The effects of this protein on pea aphids (*Acyrthosiphon pisum*) have also been investigated [16,17]. Fuchs et al. [18] reported that *Xenorhabdus szentirmaii* is a unique source of highly efficient antimicrobial peptides against almost all known plant pathogens. At present, EPNs have been identified in several geographical areas, including approximately 100 species of *Steinernema* and 26 species of *Heterorhabditis* [19,20,21,22,23,24]. However, the diversity and application of EPNs and their symbionts have not been thoroughly studied in several countries, including Saudi Arabia. Therefore, many species and strains of such potential control organisms must be evaluated to identify new biological control agents. In 2018, Noureldeen [25] isolated an EPN species, *Steinernema* sp., from the rhizosphere of pomegranate trees in Taif, Saudi Arabia and evaluated the genetic diversity among the Saudi Arabian and Egyptian EPNs’ genotypes via RAPD and ISSR markers. 

Pomegranate cultivation and its potential yield and quality are affected by many insect pests. The most problematic insect pests for pomegranates are aphids, caterpillars, fruit flies, and leaf-footed bugs [26,27]. The pomegranate butterfly, *Deudorix* (= *Virachola*) *livia* (Lepidoptera: Lycaenidae), and the carob moth, *Ectomyelois ceratoniae* (Lepidoptera: Pyralidae), are major Lepidopteran insect pests of pomegranate. Their larvae feed on the seeds, causing serious damage in many countries, including Saudi Arabia [28], Iran [29], Tunisia [30], Egypt [31], Oman [32,33], and Jordan [34]. *Virachola livia* is also reported as a pest of the date palm in Morocco [35], Saudi Arabia, Egypt, and Tunisia. In Tunisia, the yearly damage rate caused by *E. ceratoniae* to pomegranate fruits ranges from 29–72% [36], whereas the economic loss caused by this pest in Iran is 30–80% [29]. Because the larval activity of both insects is unknown, commercial insecticides are not efficient against them [37]. When pomegranates are heavily treated with pesticides, the pesticide residues are mainly concentrated in the fruits. Given that these fruits are consumed raw, contamination with pesticides is undesirable [38]. The objectives of this study were to isolate and identify EPNs and their associated symbiotic bacteria from Taif, Saudi Arabia, and to evaluate the activity of EPNs/EPBs against the pomegranate butterfly (*V. livia*) and the carob moth (*E. ceratoniae*) under laboratory conditions. 

## 2. Materials and Methods

### 2.1. Sampling and Isolation of Entomopathogenic Nematodes (EPNs)

Taif is a high-altitude region in Saudi Arabia, which includes high mountains, agricultural plates, and valleys, expected to be rich in EPN and EPB fauna because of an abundance of insect hosts. In this study, a total of 320 soil samples were randomly collected from 10 soil localities in the Taif governorate, either from cultivated sites with pomegranate, grapevine, mango, apple, fig, citrus, and rose crops, or uncultivated ones. At each site, 32 soil samples (500 g each) were collected using a hand shovel, placed into a plastic container, labelled with vegetation and date, and then transferred under ambient temperature to the laboratory for the isolation of EPNs. The soil collection process was performed as described by Khashaba et al. [39]. The soil samples were processed using a baiting technique [40] with *Galleria mellonella* larvae followed by a modified White trap [41] to isolate EPNs. The infective juvenile stages (IJs) of the EPNs emerged from the *G. mellonella* cadavers and moved to water. The IJs were collected daily in a culture flask, kept in distilled water, and stored at 15 °C in a refrigerator. To increase the population size and confirm the pathogenicity of EPNs, a fresh *G. mellonella* larva was infected with IJs. The isolated nematodes were morphologically identified as *Steinernema* spp. or *Heterorhabditis* spp. by examining the morphometrics of the IJs and first-generation males [42].

### 2.2. Isolation of EPN-Associated Microbiota

Symbiotic bacteria associated with EPNs were isolated from the haemolymph of dead *G. mellonella* larvae, which had been infected with the IJs of EPNs according to the method of Poinar and Thomas [43], as modified by Vitta et al. [44]. In summary, the dead larvae of *G. mellonella* were surface sterilised via washing with absolute ethanol for 1 min and placed in a sterile Petri dish to dry. Subsequently, the 3rd segments from the heads of *G. mellonella* larvae were incised by a sterile sharp needle to create an influx of the haemolymph containing symbiotic bacteria. A sterile loop was used to distribute and streak the haemolymph samples on NBTA medium (nutrient agar with 0.004% triphenyl tetrazolium chloride and 0.025% bromothymol blue) and incubated at 28 °C for 48 h [14]. Bacteria were routinely grown every 24 h until pure isolated colonies were obtained. A single colony of each isolate of symbiotic bacteria was inoculated in 5 mL of Luria–Bertani (LB) broth and incubated with shaking at 220 rpm overnight at 28 °C. For the bioassay experiments, the isolated bacterial cultures in LB broth were diluted in sterile distilled water to an OD_600_ of 0.1 with a spectrophotometer. Finally, the resulting bacterial cell suspension concentration was adjusted to 1 × 10^8^ colony-forming units (CFU) per mL. The bacteria were then stored at −80 °C with 20% glycerol (*v/v*) for further study.

### 2.3. Identification of EPN-Associated Bacteria

In order to characterize the isolates, we selected only Isolate4 and Isolate16 for further molecular characterization. The genomic DNA of isolated bacteria was extracted from bacterial pellets using a bacteria genomic DNA miniprep kit (QIAprep Spin Miniprep Kit). The genomic DNA of bacteria was stored at −20 °C for further use in PCR. PCR-based analysis and 16S rRNA gene sequencing were performed to identify bacterial species [45] using the inter-universal primers 785F (GGATTAGATACCCTGGTA) and 907R (CCGTCAATTCMTTTRAGTTT). 

### 2.4. Molecular Characterisation through Phylogenetic Tree Analysis

The 16Sr RNA sequence of each isolate was blast against the NCBI “16S ribosomal RNA sequence (Bacteria and Archaea)” database, and the top five targets were collected for further phylogenetic tree analysis. All 16S sequences were aligned using the MUSCLE program with 50 iterations. The aligned sequences were displayed in a CLC viewer. The evolutionary history was inferred by using the maximum likelihood method based on the Tamura–Nei model [46]. The tree with the highest log likelihood (-1426.34) is shown. The percentage of trees in which the associated taxa clustered together is shown next to the branches. Initial tree(s) for the heuristic search were obtained automatically by applying the Neighbor-Join and BioNJ algorithms to a matrix of pairwise distances estimated using the maximum composite likelihood (MCL) approach and then selecting the topology with a superior log-likelihood value. The tree is drawn to scale, with branch lengths measured in the number of substitutions per site. All positions containing gaps and missing data were eliminated. There were a total of 242 positions in the final dataset. Evolutionary analyses were conducted using MEGA7 [47] with 1000 bootstrap values.

### 2.5. Insects

Following methods by Alotaibi et al. [48], pomegranate butterfly (*V. livia*) and carob moth (*E. ceratoniae*) larvae were collected from commercial pomegranate orchards located in Taif, Saudi Arabia, and then reared on a wheat bran diet (300 g of wheat bran, 50 g of sugar, 120 mL of water, 130 mL of glycerol, and 9 g of yeast), whereas adults were fed with a 10% honey–water solution. The rearing room was adjusted to 25 ± 1 °C, a relative humidity of 60% and a light–dark cycle of 16 L:8 D.

### 2.6. Bioassays

#### 2.6.1. Pathogenicity of EPNs

Five third instar larvae of the pomegranate butterfly or carob moth were placed individually in 5-cm-diam. Plates lined with a Whatman’s No. 2 filter paper. One millilitre of EPN suspension from each species (*Steinernema* spp. and *Heterorhabditis* spp.) was added directly by micropipette at rates of 160, 80, 40, 20, 10, and 5 IJs per insect larva. Five grams of pomegranate seeds were provided as food for the larvae. In the control treatment, 1 mL of distilled water was added to the plate. The plates were then covered and incubated in a controlled growth chamber under the same conditions described above. Subsequently, *V*. *livia* and *E. ceratoniae* larval mortalities were monitored daily for up to 4 days, and the dead larvae were dissected under the stereomicroscope to confirm the infections. Each treatment was replicated 5 times, and the entire experiment was conducted twice.

#### 2.6.2. Pathogenicity of EPBs

In this experiment, two isolates of bacterial associations were selected for use in the bioassay. The bacteria, including *Pseudomonas mosselii* SJ10 associated with *Heterorhabditis* spp. and *Stenotrophomonas maltophilia* strain CQ1 associated with *Steinernema* spp., were used to determine the oral toxicity against *V*. *livia* and *E. ceratoniae* larvae. In summary, 5 g of pomegranate seeds were immersed in 2 mL of each bacterial suspension at concentrations of 1 × 10^8^, 1 × 10^6^, 1 × 10^4^, and 1 × 10^2^ CFU/mL for 30 sec. The treated seeds were then picked up and placed in a plastic plate (5 cm) lined with filter paper (Whatman number 2). Then, 5 *V*. *livia* or *E. ceratoniae* larvae were put into the plastic plate, which was then covered and incubated as previously described. Equal proportions of distilled water were used as controls. Finally, the mortality rates of *V*. *livia* and *E. ceratoniae* larvae were recorded for 6, 12, 24, and 72 h following treatment. Each bioassay was repeated twice in five replicates, each on different dates. 

### 2.7. Statistical Analysis

The data obtained are expressed in terms of mean ± standard error (M ± SE). Percente larval mortality was analysed using 2-way analysis of variance (ANOVA), with IJ rate or CFU concentration and exposure duration as independent factors, followed by Duncan’s multiple range test. All mortality data analyses were conducted using the Costat program (Version 6.45). Furthermore, data for LC_50_ and LC_90_ values were subjected to chi-square tests for both EPNs and EPBs using SPSS Version 23 (*p* < 0.05), in which *p*-values less than 0.05 indicate statistical significance.

## 3. Results

### 3.1. Isolation of Entomopathogenic Nematodes from Soil Samples

Out of the 10 soil sites from Taif Province, Saudi Arabia, seven tested positive for EPNs (Table 1). The positive sites were Thomala, Al-Haweyia, Bani-Malik, Al-Shafa, Taif University, Alsail Alkabir, and Garoah. Soil samples recovered from the Thomala region revealed the largest number of EPNs (10), followed by Al-Haweyia (7) and Al-Shafa (6). Of the 32 soil samples collected from Bani-Malik, five EPN isolates were recorded, whereas Taif University gardens revealed the presence of three EPN isolates. Moreover, two EPNs were present in the soil of two sites: Alsail Alkabir and Garoah (Table 1). Data presented in Table 2 show that out of the eight vegetation types examined, six were positive for *Steinernema* and five for *Heterorhabditis* isolates. Among the 320 soil samples, 35 (10.9%) were positive for *Steinernema* or *Heterorhabditis* (Table 2). This yielded 15 isolates (4.69%) of *Heterorhabditis* and 20 isolates (6.25%) of *Steinernema*. Most EPNs were isolated from the soil of the citrus rhizosphere (13 isolates), followed by the pomegranate (9 isolates) and the grapevine (7 isolates), with occurrence rates of 32.5%, 22.5% and 17.5%, respectively. Two EPN isolates were found in the rhizosphere soil of fig and mango vegetation, with occurrence rates of 5% each, whereas only one EPN isolate was recorded from rose and apple crop soil. Furthermore, none of the EPNs were isolated from the uncultivated soil (Table 2).

### 3.2. Isolation and Identification of EPBs through 16S rRNA Gene Sequencing 

**Figure 1:**
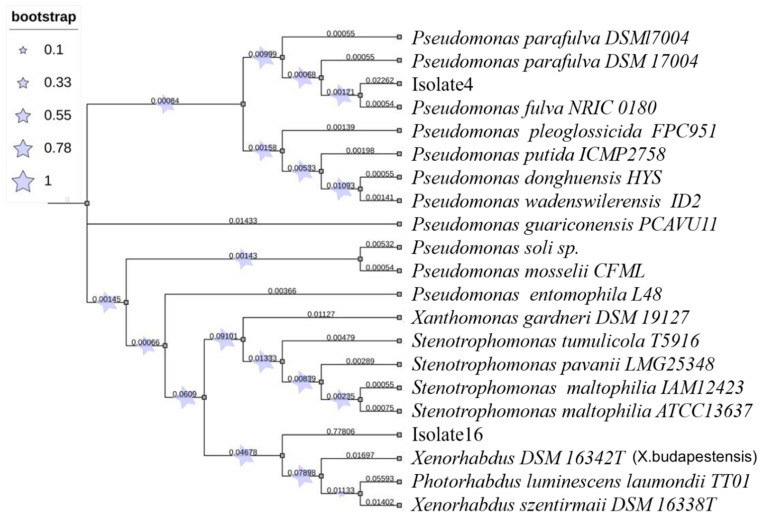
Phylogenetic tree representing distances based on the 16S rRNA sequences acquired from isolates within insect pathogenic nematodes that infected pomegranate plants.

Based on colony morphology on NBTA agar, 26 isolates of EPN-associated bacteria were identified as *Xenorhabdus* (6 isolates), *Photorhabdus* (4 isolates), *Pseudomonas* (7) or *Stenotrophomonas* (9). The molecular characterization of Isolate4 and Isolate16 also validated our morphological identification as *Pseudomonas* sp. and *Xenorhabdus* sp. For instance, the Isolate4 showed a 99.419% identity match with *Pseudomonas soli* strain F-279 (NR_134794.1), *Pseudomonas mosselii* strain CFML 90-83 (NR_024924.1) and *Pseudomonas entomophila* L48 (NR_115336.1) (Figure 1). Similarly, Isolate16 showed identity (99% similarity) with *Xenorhabdus* sp. The phylogenetic tree analysis also confirmed that Isolate4 belonged to *Pseudomonas* sp., and Isolate16 belonged to *Xenorhabdus* sp.

### 3.3. Pathogenicity Assays

#### 3.3.1. Virulence of EPNs

**Figure 2:**
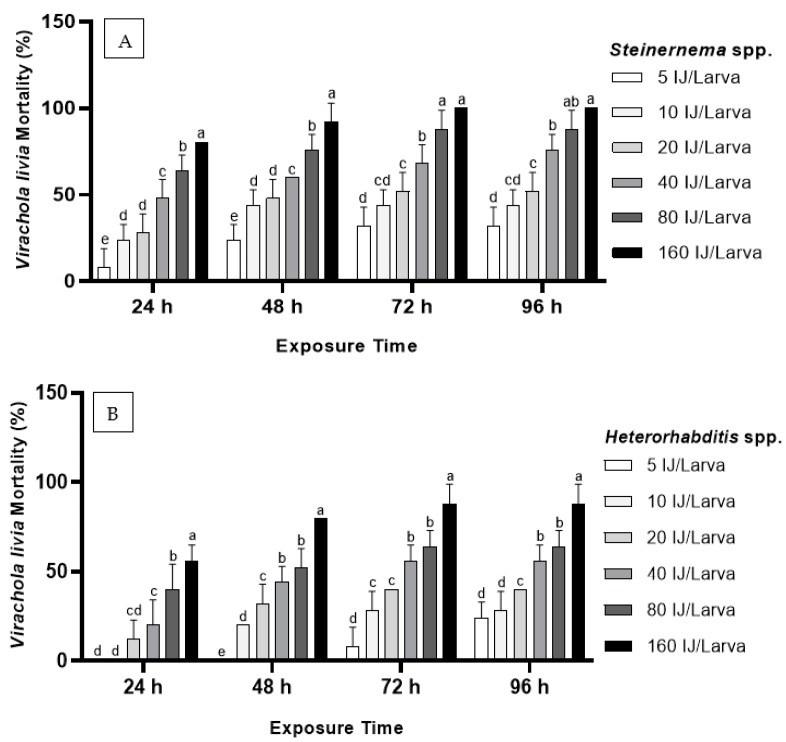
Mortality percentage (mean ± SE) of the pomegranate butterfly, *Virachola livia*, exposed to different levels (5, 10, 20, 40, 80, and 160 IJ/Larva) of *Steinernema* spp. (**A**) and *Heterorhabditis* spp. (**B**) at different exposure times (24, 48, 72, and 96 h). Bars annotated with the same letter are not significantly different (*p* < 0.05, based on Duncan test).

The insecticidal activity of the isolated EPNs and their associated bacteria against insect hosts was further evaluated to determine their capacity to be used as biological control agents. The data in Figure 2, A and B show that both *Steinernema* spp. and *Heterorhabditis* spp. had a highly significant effect on the mortality of *V*. *livia* larvae (*p* < 0.05). The results show that both nematode species increased mortality of *V*. *livia* larvae (*p* < 0.05) when compared to the control treatment, which recorded zero mortality at all exposure times. The *Steinernema* spp. isolate induced 100% mortality, and the *Heterorhabditis* spp. isolate induced 88% mortality at 160 IJs/larva and 96 h post-treatment. The results also show a direct, significant relationship between the mortality rates and both IJ concentration and exposure time (*p* < 0.05). Thus, as the IJ concentration and exposure time increased, the mortality rate increased. The overall mortality of *V*. *livia* larvae after treatment with 5 to 160 IJs of *Steinernema* spp./larva ranged between 8% and 100% (Figure 2A), whereas it ranged from 0% to 88% (Figure 2B) for *Heterorhabditis* spp.

**Figure 3:**
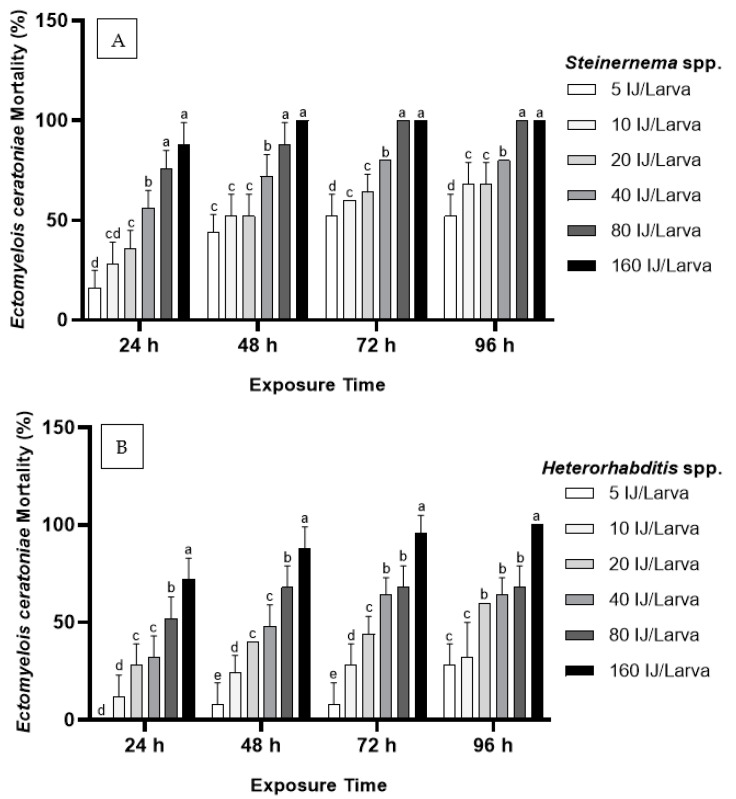
Mortality percentage (mean ± SE) of the carob moth, *Ectomyelois ceratoniae*, exposed to different levels (5, 10, 20, 40, 80, and 160 IJ/Larva) of *Steinernema* spp. (**A**) and *Heterorhabditis* spp. (**B**) at different exposure times (24, 48, 72 and 96 h). Bars annotated with the same letter are not significantly different (*p* < 0.05, based on Duncan test).

The data in Figure 3, A and B show that third instar *E. ceratoniae* larvae were highly susceptible (*p* < 0.05) to both *Steinernema* spp. and *Heterorhabditis* spp.; they exhibited 100% mortality at 96 h post treatment. According to the results, *Steinernema* spp. surpassed *Heterorhabditis* spp. in inducing mortality in *E. ceratoniae* larvae. *Steinernema* spp. induced 100% larval mortality from 48 to 96 h exposure, compared with 88%, 96% and 100% mortality induced by *Heterorhabditis* spp. at 160 IJs/larva and the same exposure times, respectively. Compared to the control (0 mortality), an increase in larval mortality with the increase of IJ concentration and exposure time was also observed (Figure 3). At 24 h post treatment, *Steinernema* spp. isolates caused larval mortality rates ranging from 16% to 88% (Figure 3A). *Heterorhabditis* spp. did not cause larval mortality at a concentration of 5 IJs/larva, and exhibited virulence (8% mortality) up to 72% when applied at a range between 10 and 160 IJs/larva (Figure 3B).

As shown in Table 3, the data indicate that *Steinernema* spp. was significantly more efficient against *V. livia* larvae than *Heterorhabditis* spp. under laboratory conditions. The *Steinernema* spp. isolate exhibited lower LC_50_ and LC_90_ values of 43 and 352.8 IJs/larva, respectively, at 24 h; 18.5 and 208.1 IJs/larva, respectively, at 48 h; 13.6 and 97.9 IJs/larva, respectively, at 72 h; and 12.9 and 85.5 IJs/larva, respectively, at 96 h. However, for the *Heterorhabditis* spp. isolate, the LC_50_ and LC_90_ values were 118 and 586.2 IJs/larva, respectively, at 24 h; 52 and 359.6 IJs/larva, respectively, at 48 h; and 32.4 and 243.9 IJs/larva, respectively, at 72 h. At 96 h, it exhibited LC_50_ and LC_90_ values of 28.3 and 226 IJs/larva, respectively (Table 3).

Likewise, the data in Table 4 reveal that the *Steinernema* spp. isolate was more effective against *E. ceratoniae* than the *Heterorhabditis* spp. isolate, with LC_50_ values at 24, 48, 72, and 96 h after treatment of 28.1, 9, 6.6, and 5.7 IJs/larva, respectively, for the former and 68.3, 34.4, 26.6, and 20.2 IJs/larva, respectively, for the latter. Similar results were obtained for the LC_90_ values, which were significantly lower for *Steinernema* spp. than for *Heterorhabditis* spp. Furthermore, the data in Table 4 show that the highest degree of homogeneity in the *E. ceratoniae* larval response was observed after exposure to the *Heterorhabditis* spp. isolate, with a slope value of 0.119 at 72 h post-treatment. In contrast, the other tested IJ levels exhibited low slope values (Table 4), which indicates heterogeneity in the response of *E. ceratoniae* larvae to these levels.

#### 3.3.2. Virulence of EPN-Associated Bacteria

The data in Table 5 represent the toxicity of two species of bacteria—*Pseudomonas mosselii* SJ10 (associated with the EPN *Heterorhabditis* spp.) and *Stenotrophomonas maltophilia* CQ1 (associated with *Steinernema* spp.)—against *V. livia* larvae under laboratory conditions. Both EPB species significantly affected larval mortality (*p* < 0.05); however, percent larval mortality caused by *S. maltophilia* CQ1 (78.5%) was significantly higher than that caused by *P. mosselii* SJ10 (66%). The percentage of larval mortality increased significantly with bacterial cell concentration and exposure duration (*p* < 0.05). The interactive effect of EPB species, cell concentrations, and exposure time on larval infection was not significant (*p* = 0.9742). The highest mortality percentage (100%) was recorded in the plates where the larvae were exposed to *S. maltophilia* CQ1 at the rate of 10^8^ CFU/mL distilled water after 24 and 48 h of application, and the lowest (32%) was recorded when the larvae were exposed to 10^2^ CFU/mL of *P. mosselii* SJ10 6 h post-treatment (Table 5).

The data in Table 6 show the toxicity levels of the EPBs. *S. maltophilia* CQ1 was more effective than *P. mosselii* SJ10. After 6 and 12h of exposure against *V. livia* larvae, *S. maltophilia* CQ1 exhibited LC_50_ values of 1.26 × 10^2^ and 5.01 × 10^1^ CFU/mL, respectively, whereas *P. mosselii* SJ10 exhibited LC_50_ values of 1.26 × 10^4^ and 7.94 × 10^2^, respectively. However, at 48 h of exposure, no significant difference was detected between the LC_50_ values of these bacterial species. *Stenotrophomonas maltophilia* CQ1 exhibited LC_50_ value of 6.31 CFU/mL compared with 10 CFU/mL for *P. mosselii* SJ10. It is also clear that both EPBs exhibited high slope values (more than 2), which indicates homogeneity in the response of *V. livia* larvae to these bacteria (Table 6).

Similarly, as shown in Table 7 and Table 8, both *S. maltophilia* CQ1 and *P. mosselii* SJ10 bacteria successfully induced mortality in *E. ceratoniae* larvae (*p* < 0.05). The data also indicate that the mortality rate had a direct relationship with the exposure time and bacterial CFU concentration (*p* < 0.05). The regression analysis of the data shows that the mortality of *E. ceratoniae* larvae significantly increased with increasing bacterial concentration (R^2^ = 0.9743; *p* < 0.05). Maximum (58.7%) and minimum (38.7%) mortality rates were achieved when the larvae were treated with 10^8^ and 10^2^ CFU/mL, respectively. *Stenotrophomonas maltophilia* CQ1 was more virulent than *P. mosselii* SJ10; it induced 82.3% mortality in third instar *E. ceratoniae* larvae compared with 71.3% for *P. mosselii* SJ10. The maximum larval mortality rate was caused by *S. maltophilia* CQ1 at a concentration of 10^8^ CFU/mL (100%) after 24 and 48 h of application, whereas the minimum was recorded in the larvae treated with the isolate *P. mosselii* SJ10 at 10^2^ CFU/mL (40%) after 6 h (Table 7). The calculated LC_50_ and LC_90_ values obtained with *S. maltophilia* CQ1 at 24 and 48 h after treatment against *E. ceratoniae* were 9.33 and 1 × 10^4^ CFU/mL and 4.79 and 1 × 10^3^ CFU/mL, respectively, whereas they were 15.8 and 2.51 × 10^9^ CFU/mL and 10 and 7.94 × 10^5^ CFU/mL, respectively, for *P. mosselii* SJ10 (Table 8). Furthermore, the highest degree of homogeneity for the larvae of *E. ceratoniae* was observed under exposure to *S. maltophilia* CQ1, with a slope value of 2.8.

## 4. Discussion

The present investigation showed a 10.94% rate of recovery of EPNs in the soil samples from the Taif region of Saudi Arabia. These results may be attributed to soil parameters (e.g., temperature, pH, and moisture), which are important for EPN survival and infectivity. The isolated EPNs were only identified to the genus level in the present study; thus, future research should identify them to species level. 

Our findings are in accordance with those reported by Noureldeen [25], who recorded the occurrence of one EPN species, *Steinernema* sp., in the rhizosphere of pomegranate trees in Taif, Saudi Arabia. Likewise, EPNs have been identified in several geographical areas including approximately 100 species of *Steinernema* and 26 species of *Heterorhabditis* [22,24]. Of the symbiotic bacteria, the morphologically identified *Xenorhabdus* species (isolate16) was molecularly characterized as *Xenorhabdus budapestensis* or *Xenorhabdus szentirmaii*, whereas the isolate4 was molecularly identified as *Pseudomonas fulva*. In addition, in the present study, we found that the bacteria *S. maltophilia* CQ1 was associated with *Steinernema* spp., whiles the bacteria *P. mosselii* SJ10 was symbiotically associated with *Heterorhabditis* spp., which are reported for the first time in Saudi Arabia. These results are consistent with those previously recorded by Ogier et al. [49], who mentioned that the association between *Steinernema* and *Xenorhabdus* was never monoxenic. Therefore, the bacterial community associated with laboratory-reared IJs from the *Steinernema carpocapsae*, *S. feltiae*, *S. glaseri,* and *S. weiseri* EPN species consisted of several Proteobacteria. The authors also reported that the laboratory-reared IJs of *S. carpocapsae* had a bacterial community composed of the core symbiont *Xenorhabdus nematophila*, together with a frequently associated microbiota (FAM) consisting of about a dozen Proteobacteria (*Pseudomonas*, *Stenotrophomonas*, *Alcaligenes*, *Achromobacter*, *Pseudochrobactrum*, *Ochrobactrum*, *Brevundimonas*, *Deftia*, etc.) [49]. Two species, *Pseudomonas protegens* and *P. chlororaphis*—the FAM members potentially involved in the parasitic lifecycle of *Steinernema*—displayed entomopathogenic properties suggestive of a role in *Steinernema* virulence and involvement in the *Steinernema* pathobiome. Indeed, in our study, the isolated bacteria were frequently detected in the haemolymph of insects infected with *Steinernema* and *Heterorhabditis*. It is generally assumed that non-symbiotic bacteria randomly “hitchhiked” in the IJ vectors via the cuticle or intercuticular space are introduced into the insect haemocoel during IJ penetration [50]. For example, *Stenotrophomonas*, *Ochrobactrum*, and *Pseudomonas* have often been identified in the soil-dwelling *Caenorhabditis elegans* nematodes [51]. Likewise, Proteobacteria is the most abundant phylum in bacterial communities associated with plant roots [52], as well as in plant-covered soils, such as the rhizosphere [53].

Regarding their insecticidal activity, our investigations show that *Steinernema* spp. were more effective than *Heterorhabditis* spp. against *V. livia* and *E. ceratoniae* larvae under laboratory conditions. These results are consistent with those of Memari et al. [54], who observed that mortality rates of *E. ceratoniae* larvae in laboratory tests corresponded to LC_50_ values of 2.02 IJ/larva for *S. feltiae* and 2.05 IJ/larva for *S. carpocapsae*. On the contrary, *H. bacteriophora* showed low virulence against the larvae, with an LC_50_ of 426.92 IJ/larva [54]. Our findings also show that *E. ceratoniae* larvae have higher susceptibility to both EPN species tested than *V. livia*, which may be due to the strong virulence of EPNs on members of the Pyralidae family such as the wax moth (*G. mellonella*) larvae. Furthermore, because *E. ceratoniae* overwinters throughout the autumn and winter seasons as larvae within infested fruit that drop on the soil, we hypothesise that it is possible to use EPNs for their control. Although most of the Steinernematids have ambush behaviours, the success of our tested species could be attributed to the mobile behaviours of *V. livia* and *E. ceratoniae* larvae, which may increase the distribution patterns of *Steinernema* spp. and can thus, increase the pest’s mortality [55]. For instance, Christos et al. [56] also reported the potential of three concentrations (100, 300 and 900 IJs/larva) of *S. feltiae* for the control of the Mediterranean flour moth, *Ephestia kuehniella* (Lepidoptera: Pyralidae), in stored wheat. 

The larvicidal activity of the two EPN-associated bacterial species was also evaluated against the two pomegranate pests in the current study. It is clear that the bacteria *S. maltophilia* CQ1 and *P. mosselii* SJ10 have the capacity to control *V. livia* and *E. ceratoniae* larvae. Our data also revealed that *S. maltophilia* CQ1 was more effective than *P. mosselii* SJ10 against both *V. livia* and *E. ceratoniae* larvae; however, *E. ceratoniae* was more susceptible. The higher lethality of *S. maltophilia* CQ1 (that is associated with *Steinernema* spp.) in comparison to *P. mosselii* SJ10 (that is associated with *Heterorhabditis* spp.) correlates with the greater lethality of *Steinernema* spp. over *Heterorhabditis* spp. on *V. livia* and *E. ceratoniae* larvae. These results are consistent with those of Alotaibi et al. [48], who recorded that mortality of *E. ceratoniae* larvae caused by two *Xenorhabdus* bacterial species was significantly higher than that of *Photorhabdus* species. In contrast, a recent study by Elbrense et al. [15] showed that *H. bacteriophora* and its symbiont, *Photorhabdus* sp., were more virulent than *S. riobravis* and its symbiont, *Xenorhabdus* sp. against *Pieris rapae* and *Pentodon algerinus*. Our results are also consistent with those of Jabeen et al. [57], who quantified bacterial chitinase production by *S. maltophilia* and their termiticidal activity. Moreover, this study concurred with Amer et al. [58], who stated that *S. maltophilia* shows promising antagonistic activity against a panel of multidrug-resistant bacteria and fungi. Similarly, Berg [59] reported that *S. maltophilia* inhibited the growth of phytopathogen *Rhizoctonia solani*, possibly because of antibiosis and the production of some lytic enzymes that act against pathogenic fungi. Subsequent studies have revealed that the metabolic diversity of *S. maltophilia* is responsible for the production of novel bioactive compounds, including agents that can be used in biocontrol against microorganisms and insects [60]. 

## 5. Conclusions

In this study, EPNs and their associated bacteria were isolated and identified from Taif, Saudi Arabia, and their biocontrol efficacy was evaluated against the third instar larvae of *V. livia* and *E. ceratoniae*, two important insect pests of pomegranate. *Steinernema* spp. were more virulent than *Heterorhabditis* spp. against the two pomegranate insects. In addition, the bacteria *Stenotrophomonas maltophilia* CQ1, isolated from *Steinernema* spp., surpassed those of the bacteria *Pseudomonas mosselii* SJ10, associated with *Heterorhabditis* spp., in their ability to kill *V. livia* or *E. ceratoniae* larvae. We conclude that either application of either EPNs’ IJs or their associated EPBs could serve as potential biocontrol agents for *V. livia* and *E. ceratoniae* for sustainable agriculture. These studies were conducted under laboratory conditions; thus, future studies need to validate these results under field conditions.

## Figures and Tables

**Table 1:** Characterization of the vegetation and the sampling sites.

Location	Vegetation	Entomopathogenic Nematodes
Thomala (32) *	Citrus (6) *, Grapevine (7), Pomegranate (10), Uncultivated (9)	+ (10) **
Al-Haweyia (32)	Fig (8), Mango (7), Citrus (10), Grapevine (7)	+ (7)
Al-Mathnah (32)	Grapevine (13), Fig (9), Pomegranate (10)	-
Liyah (32)	Mango (13), Apple (12), Uncultivated (7)	-
Bani-Malik (32)	Citrus (10), Grapevine (7), Pomegranate (10), Uncultivated (5)	+ (5)
Al-Hada (32)	Grapevine (6), Uncultivated (9), Roses (17)	-
Al-Shafa (32)	Citrus (9), Uncultivated (6), Apple (17)	+ (6)
Taif University (32)	Pomegranate (5), Citrus (5), Roses (19), Uncultivated (3)	+ (3)
Alsail Alkabir (32)	Apple (11), Pomegranate (5), Fig (11), Roses (4), Uncultivated (1)	+ (2)
Garoah (32)	Fig (12), Mango (20)	+ (2)

* Numbers between parentheses are the number of collected samples from each site and vegetation. ** Number between parentheses indicates the number of EPNs isolates recovered from each positive site.

**Table 2:** Occurrence of EPNs isolated from the rhizosphere of vegetation at Taif governorate, Saudi Arabia.

	EPNs	*Steinernema* sp.	*Heterorhabditis* sp.	Total of EPNs Isolates	Frequency of Occurrence %
Vegetation					
Citrus (40)		+ (7) *	+ (6)	2 (13)	32.5
Pomegranate (40)		+ (5)	+ (4)	2 (9)	22.5
Grapevine (40)		+ (4)	+ (3)	2 (7)	17.5
Fig (40)		+ (2)	-	1 (2)	5
Apple (40)		-	+ (1)	1 (1)	2.5
Mango (40)		+ (1)	+ (1)	2 (2)	5
Roses (40)		+ (1)	-	1 (1)	2.5
Uncultivated (40)		-	-	0	0
Total of positive vegetation		6 (20)	5 (15)	11 (35)	
Frequency of occurrence %		6.25	4.69	10.94	

* Numbers between parentheses are the number of positive samples.

**Table 3:** Pathogenicity of two EPNs against the pomegranate butterfly, *Virachola livia*.

EPNs	Exposure Time (h)	LC_50_ IJ/Larva (95% LCL–UCL)	LC_90_ IJ/Larva (95% LCL–UCL)	Slope ± SE	Intercept	*X* ^2^	*p*-Value
*Steinernema* spp.	24	43 (29.8–66.3)	352.8 (179.6–1254.9)	0.11 ± 0.02	4.94	0.74	0.007
	48	18.5 (11.2–27.9)	208.1 (106.3–793.4)	0.095 ± 0.019	9.4	1.56	0.008
	72	13.6 (8.6–19.3)	97.9 (59.6–238)	0.103 ± 0.021	10.6	3.47	0.007
	96	12.9 (8.2–18.2)	85.5 (53.5–194.2)	0.101 ± 0.024	11.01	2.70	0.014
*Heterorhabditis* spp.	24	118 (82.8–210.7)	586.2 (295.2–2421.9)	0.092 ± 0.012	0.49	1.58	0.002
	48	52 (37–79.9)	359.6 (189.7–1177.3)	0.108 ± 0.022	3.8	3.87	0.008
	72	32.4 (22.7–47.4)	243.9 (134.3–720.1)	0.109 ± 0.024	6.1	1.75	0.011
	96	28.3 (19.4–41.3)	226 (124.2–674.6)	0.105 ± 0.025	7	0.80	0.014

LC_50_—lethal concentration that kills 50% of insects; LC_90_—lethal concentration that kills 90% of insects; LCL—lower confidence limit; UCL—upper confidence limit; *X*^2^—chi-square value; SE—standard error; *p*-value—probability.

**Table 4:** Pathogenicity of two EPNs against the carob moth, *Ectomyelois ceratoniae*.

EPNs	Exposure Time (h)	LC_50_ IJ/Larva (95% LCL–UCL)	LC_90_ IJ/Larva (95% LCL–UCL)	Slope ± SE	Intercept	*X* ^2^	*p*-Value
*Steinernema* spp.	24	28.1 (19.4–40.8)	217.5 (120.8–628.7)	0.11 ± 0.023	6.7	0.64	0.009
	48	9 (4.3–14.1)	97.7 (54.9–311.2)	0.085 ± 0.015	12.9	4.17	0.005
	72	6.6 (3.1–10.2)	51.4 (32.2–123.8)	0.077 ± 0.021	14.9	5.87	0.021
	96	5.7 (2.3–9.1)	47 (29.3–116)	0.07 ± 0.02	15.8	5.4	0.026
*Heterorhabditis* spp.	24	68.3 (48.2–110.2)	451.9 (230.4–1622.4)	0.104 ± 0.018	2.7	2.30	0.004
	48	34.4 (24.4–49.9)	237.1 (133.7–656.5)	0.114 ± 0.022	5.5	1.18	0.007
	72	26.6 (19.1–36.6)	151.5 (94–338.7)	0.119 ± 0.028	6.6	3.31	0.014
	96	20.2 (13.8–28.4)	138.1 (82.9–338.1)	0.102 ± 0.022	9.3	6.9	0.01

LC_50_—lethal concentration that kills 50% of insects; LC_90_—lethal concentration that kills 90% of insects; LCL—lower confidence limit; UCL—upper confidence limit; *X*^2^—chi-square value; SE—standard error; *p*-value—probability.

**Table 5:** Larvicidal activity of two bacterial species cells on the pomegranate butterfly, *Virachola livia*, under laboratory conditions.

Bacterial Species	Concentration(CFU/mL)	* Mortality %				Bacterial Species Means
		6 h	12 h	24 h	48 h	
*Pseudomonas mosselii*	10^2^	** 32 ± 8 j	48 ± 4.9 i	56 ± 7.5 ghi	64 ± 7.5 efg	66 b
	10^4^	52 ± 4.9 hi	60 ± 9 fgh	68 ± 4.9 ef	80 ± 0 cd	
	10^6^	60 ± 6.3 fgh	72 ± 4.9 de	80 ± 0 cd	84 ± 4 bc	
	10^8^	64 ± 9.8 efg	68 ± 4.9 ef	80 ± 6.3 cd	88 ± 4.9 bc	
*Stenotrophomonas maltophilia*	10^2^	48 ± 4.9 i	52 ± 4.9 hi	56 ± 7.5 ghi	72 ± 4.9 de	78.5 a
	10^4^	72 ± 4.9 de	80 ± 0 cd	84 ± 4 bc	88 ± 4.9 bc	
	10^6^	72 ± 4.9 de	80 ± 0 cd	88 ± 4.9 bc	92 ± 4.9 ab	
	10^8^	84 ± 4 bc	88 ± 4.9 bc	100 ± 0 a	100 ± 0 a	
Control		0 ± 0 k	0 ± 0 k	0 ± 0 k	0 ± 0 k	0 c
Exposure Time Means		40.3 d	45.7 c	51 b	55.7 a	

* Each treatment in this experiment was represented by five replicates, each with five larvae insects. ** Numbers in each column indicate mortality ± standard error. Means with different letters within the same column or row differ significantly (*p* < 0.05 using Duncan’s Multiple Range Test).

**Table 6:** Lethal concentrations of *Pseudomonas mosselii* and *Stenotrophomonas maltophilia* on the pomegranate butterfly, *Virachola livia*, under laboratory conditions.

Bacterial Species	Exposure Time (h)	LC_50_ CFU/mL (95% LCL–UCL)	LC_90_ CFU/mL (95% LCL–UCL)	Slope ± SE	Intercept	*X* ^2^	*p*-Value
*Pseudomonas mosselii*	6	* 4.1 (1.7–7.2)	15 (13–17.9)	2.0 ± 0.42	2.6	0.123	0.019
	12	2.9 (0.5–4.4)	13.3 (10.4–15.6)	2.1 ± 0.61	4.2	0.087	0.044
	24	1.5 (0–2.8)	11.3 (7.7–13.2)	2.3 ± 0.74	5	0.202	0.052
	48	1 (0–2.2)	9.4 (5.4–11.5)	2.5 ± 0.88	6	0.056	0.069
*Stenotrophomonas maltophilia*	6	2.1 (0.3–3.2)	13.4 (7.5–15.8)	2.4 ± 0.65	4.2	0.612	0.034
	12	1.7 (0.3–2.7)	9.1 (5.8–14)	2.6 ± 0.75	4.8	0.689	0.043
	24	1.3 (0.2–2.1)	5 (3.6–8.4)	2.8 ± 0.87	6	2.04	0.051
	48	0.8 (0–1.7)	3.9 (1.9–6.5)	2.7 ± 1.1	7.4	1.49	0.089

LC_50_—lethal concentration that kills 50% of insects; LC_90_—lethal concentration that kills 90% of insects; LCL—lower confidence limit; UCL—upper confidence limit; *X*^2^—chi-square value; SE—standard error; *p*-value—probability. * Each figure is represented as a power of 10.

**Table 7:** Larvicidal activity of two bacterial species cells on the carob moth, *Ectomyelois ceratoniae*, under laboratory conditions.

Bacterial Species	Concentration (CFU/mL)	* Mortality %				Bacterial Species Means
		6 h	12 h	24 h	48 h	
*Pseudomonas mosselii*	10^2^	** 40 ± 6.3 h	52 ± 4.9 g	60 ± 6.3 fg	68 ± 4.9 ef	71.3 b
	10^4^	60 ± 0 fg	68 ± 4.9 ef	76 ± 4 de	84 ± 4 cd	
	10^6^	60 ± 6.3 fg	76 ± 4 de	84 ± 4 cd	92 ± 4.9 abc	
	10^8^	68 ± 8 ef	76 ± 4 de	84 ± 4 cd	92 ± 4.9 abc	
*Stenotrophomonas maltophilia*	10^2^	52 ± 4.9 g	56 ± 4 g	60 ± 6.3 fg	76 ± 4 de	82.3 a
	10^4^	76 ± 4 de	84 ± 4 cd	88 ± 4.9 bc	92 ± 4.9 abc	
	10^6^	76 ± 4 de	84 ± 4 cd	92 ± 4.9 abc	96 ± 4 ab	
	10^8^	88 ± 4.9 bc	96 ± 4 ab	100 ± 0 a	100 ± 0 a	
Control		0 ± 0 i	0 ± 0 i	0 ± 0 i	0 ± 0 i	0 c
Exposure Time Means		43.3 d	49.3 c	53.7 b	58.3 a	

* Each treatment in this experiment was represented by five replicates, each with five larvae insects. ** Numbers in each column indicate mortality ± standard error. Means with different letters within the same column or row differ significantly (*p* < 0.05 using Duncan’s Multiple Range Test).

**Table 8:** Lethal concentrations of *Pseudomonas mosselii* and *Stenotrophomonas maltophilia* on the carob moth, *Ectomyelois ceratoniae*, under laboratory conditions.

Bacterial Species	Exposure Time (h)	LC_50_ CFU/mL (95% LCL–UCL)	LC_90_ CFU/mL (95% LCL–UCL)	Slope ± SE	Intercept	*X* ^2^	*p*-Value
*Pseudomonas mosselii*	6	* 2.6 (0–4.3)	13.4 (11.6–15.6)	2.1 ± 0.56	3.8	0.087	0.036
	12	1.7 (0–3.1)	11.3 (8.9–14.2)	2.2 ± 0.72	4.8	0.180	0.055
	24	1.2 (0–2.4)	9.4 (6.2–13.5)	2.4 ± 0.83	5.6	0.170	0.063
	48	1 (0.01–2)	5.9 (3.9–8.1)	2.6 ± 0.94	6.4	0.172	0.070
*Stenotrophomonas maltophilia*	6	1.8 (0.2–2.8)	10.5 (6.4–12.8)	2.5 ± 0.70	4.6	0.687	0.038
	12	1.7 (0.5–2.5)	6.2 (4.5–9.3)	2.8 ± 0.77	5	1.22	0.038
	24	0.97 (0.02–1.8)	4 (2.6–6.3)	2.7 ± 1	7	1.33	0.076
	48	0.68 (0–1.5)	3 (0.6–5)	2.7 ± 1.2	8	0.697	0.106

LC_50_—lethal concentration that kills 50% of insects; LC_90_—lethal concentration that kills 90% of insects; LCL—lower confidence limit; UCL—upper confidence limit; *X*^2^—chi-square value; SE—standard error; *p*-value—probability. * Each figure is represented as a power of 10.

## Data Availability

Not applicable.
